# Serum Biomarkers for the Diagnosis of Glaucoma

**DOI:** 10.3390/diagnostics11010020

**Published:** 2020-12-24

**Authors:** Young Joo Shin, Eunbi Kim, Bobby Kwanghoon Han, Kayoung Yi

**Affiliations:** 1 Department of Ophthalmology, Hallym University College of Medicine, Seoul 07441, Korea; schinn@hallym.or.kr (Y.J.S.); wheb8793@hanmail.net (E.K.); 2 Eyeforest Clinic, Seoul 04701, Korea; 3 Division of Rheumatology, University of Washington, Seattle, WA 98195, USA; hank4@uw.edu

**Keywords:** normal-tension glaucoma, primary open-angle glaucoma, glaucoma, biomarker, autoantibody

## Abstract

Despite the importance of the early detection of glaucoma, most patients with progressive glaucoma show minimal symptoms. We aimed to evaluate biomarkers for glaucoma diagnosis in Korea. Forty-two volunteers with/without open-angle glaucoma were enrolled from January through October 2015—divided into a control or open-angle glaucoma group, which was further divided into normal-tension glaucoma (NTG) and high-tension glaucoma (HTG) groups—and underwent assessments for myelin basic protein (MBP), heat shock protein 60, anti-Sjögren’s-syndrome-related antigen A (SSA) and antigen B (SSB), anti-α-fodrin, and anti-nucleic acid. The glaucoma group showed a higher serum MBP level and lower serum anti-α-fodrin antibody level than the control group (*p* < 0.05). The NTG group showed higher serum anti-SSA and anti-SSB levels and lower anti-α-fodrin IgG/IgA levels than the HTG group. In the receiver operating characteristic curve analysis, the area under the curve (AUC) for serum MBP level was 0.917 in discriminating between controls and patients with glaucoma. Between the NTG and HTG groups, anti-SSA, anti-SSB, and anti-α-fodrin IgG/IgA levels showed an AUC above 0.8. Thus, these biomarkers were useful for diagnosing glaucoma and discriminating between controls and patients with glaucoma, and patients with NTG and HTG.

## 1. Introduction

Glaucoma is one of the leading causes of blindness worldwide [1]. Glaucoma diagnosis is traditionally based on assessments of visual field (VF) defects or optic nerve abnormalities [2]. Early diagnosis of glaucoma is not easy because most patients with progressive glaucoma show few or no symptoms [3]. Nevertheless, early detection of glaucoma is critically important. Although the optic nerve damage in glaucoma is irreversible and progressive, early treatment can slow the disease progression [4]. Glaucoma can be classified as normal-tension glaucoma (NTG) or high-tension glaucoma (HTG). There has been a debate as to whether HTG and NTG are the same or different diseases. However, the pathogenesis [5,6], morphology [7], function [7], phenotype [8,9], and vascular associations [10] of HTG and NTG are reported to be different.

Measurement of intraocular pressure (IOP) is a common approach used to detect glaucoma [11]. High IOP remains a major risk factor for glaucoma [12], and regular optic nerve examinations can also help detect glaucoma [13]. Elevated serum nitric oxide levels and antibodies against autoantigens in ocular tissues have been recently suggested as markers in glaucoma [14,15]. Elevated and reduced serum levels of specific antibodies may contribute to the pathogenesis of glaucoma [16], facilitating the detection of glaucoma. Furthermore, serum markers of optic nerve damage identified in serological tests could also be used as indicators of glaucoma. Elevated levels of antibodies against heat shock protein 60 (HSP60), α-fodrin, Sjögren’s-syndrome-related antigen A (SSA), and Sjögren’s-syndrome-related antigen B (SSB) have been reported in patients with glaucoma [16,17]. However, these studies were mainly performed in German and American populations, where the prevalence of HTG is high. In contrast, the prevalence of NTG is high in Korea [18]. Among the 3.6% of primary open-angle glaucoma cases, 78% were NTG [19]. These racial differences may affect the usefulness of specific serum markers in the detection of glaucoma. Even though there are several studies about aqueous humor in animal models [20,21,22], there have been very few studies about using serum biomarkers, other than ferritin levels, in humans to diagnose or assess the risk of glaucoma [23]. These differences may affect the usefulness of specific serum markers in the detection of glaucoma.

In this study, we focused on the specific characteristics of biomarkers in Korean patients with glaucoma. We expect that the data obtained in this investigation will eventually facilitate the development of a new and more effective regimen for glaucoma diagnosis and treatment in Korea.

## 2. Materials and Methods

### 2.1. Design and Patients

This study was approved by the Institutional Review Board of Hallym University Medical Center and was conducted in accordance with the tenets of the Declaration of Helsinki. All participants were informed about the study, and their written consent was obtained. Volunteers with/without open-angle glaucoma were enrolled between January and October 2015. The diagnostic criteria for glaucoma were as follows: open-angle on gonioscopy and glaucomatous optic disc damage with corresponding glaucomatous changes in the VF [24]. The glaucomatous disc changes included disc notching, neuroretinal rim thinning, an enlarged cup/disc ratio, and retinal nerve fiber thickness (RNFL) defects [25,26]. Although the optic disc was evaluated as previously published [25,26], statistical processing according to severity was not performed because of the small number of participants. Patients with glaucoma showed VF defects corresponding to the RNFL defects; typically, an inferior VF defect corresponded to a superior RNFL defect. The VF was examined with the Humphrey^®^ Field Analyzer/HFA^™^ II-I Series, which yielded reliable results. The eyes showed open angles and the absence of other possible causes of optic neuropathy (e.g., infection, inflammation, meningeal disease, ischemic disease, compressive lesions). Patients with a history of intracranial lesions were excluded. The glaucoma group was divided into two subgroups: NTG and HTG. Participants in the control group did not have glaucoma and showed no clinical signs of primary or secondary glaucoma or any additional eye disease other than cataracts. The IOP in the patients with NTG was not greater than 21 mm Hg (without treatment), as determined by Goldmann applanation tonometry (GAT), while the IOP in the patients with HTG, also determined by GAT, was greater than 21 mm Hg. IOP measurement was repeated thrice on the day of blood sampling and the mean of these measurements was used in the analyses. The exclusion criteria were as follows: elevated IOP attributable to certain defined causes such as trauma, uveitis, steroid administration, or exfoliative, pigmentary, or neovascular glaucoma; and known autoimmune diseases such as Sjögren’s syndrome, scleroderma, Lupus syndrome, and insulin-dependent diabetes mellitus.

### 2.2. Blood Samples

Blood samples were collected from all volunteers after they had provided their informed consent. Whole blood (5 mL) was collected from the antecubital vein into a BD Vacutainer^®^ SST™ tube for serum (BD Diagnostics, Oxford, UK). The blood was allowed to clot by leaving it undisturbed at room temperature (RT) for 15 min. The samples were centrifuged at 1000× *g* for 15 min at 4 °C, and the resultant serum was stored at −80 °C for subsequent analysis.

### 2.3. Enzyme-Linked Immunosorbent Assay

The serum samples were maintained at −80 °C until the assay. The levels of anti-SSA antibody, anti-SSB antibody, HSP60, anti-α-fodrin antibody, myelin basic protein (MBP), and anti-nucleic acid (ANA) antibody were measured by enzyme-linked immunosorbent assay (ELISA) with the SSA IgG ELISA kit (KA0949, Abnova, Taoyuan City, Taiwan), SSB IgG ELISA kit (KA0950, Abnova), HSP60 ELISA kit (ADI-EKS-600, Enzo Life Sciences, Farmingdale, NY, USA), α-fodrin Ab IgG/IgA ELISA Kit (KA1087, Abnova), MBP ELISA kit (E-EL-H0161, Elabscience, Houston, TX, USA), and ANA Screen ELISA Kit (KA0939, Abnova), respectively, in accordance with the manufacturers’ protocols. Briefly, for measurement of serum HSP60, anti-SSA antibody, anti-SSB antibody, anti-α-fodrin IgG/IgA antibody, and ANA IgG antibody levels, 96-well microplates coated with primary antibodies were utilized. After dispensing the samples into the wells, the plate was incubated for 2 h at RT. The wells were washed, and an enzyme conjugate was dispensed into each well, which was incubated for 2 h at RT. After the enzyme conjugate was washed with a washing buffer, 3,3′,5,5′-tetramethylbenzidine substrate was dispensed, and the plate was again incubated at RT. Subsequently, stop solution was added, and optical density (O.D.) was measured at 450 nm using an ELISA reader. For assessment of the serum anti-SSA antibody, anti-SSB antibody, and ANA antibody levels, the antibody index was calculated as:

$$\mathrm{Antibody}\;\mathrm{index}\;=\;\frac{\mathrm{Sample}\;O.D.}{\mathrm{Cut}-\mathrm{off}\;\mathrm{Value}}$$

The cut-off value was calculated as:

Cut−off Value =Calibrator mean O.D.×Calibrator Factor (0.5)

For measurement of serum MBP levels, the samples were added to the pre-coated plate wells and incubated at 37 °C. After removing the liquid from each well, a biotinylated detection antibody solution was added. After incubating the plate again at 37 °C, horseradish peroxidase conjugate working solution was added to each well. The plate was incubated once more at 37 °C, and the substrate solution was added to each well. Subsequently, the stop solution was added to each well, and the O.D. of each well was then determined at 450 nm using a microplate reader.

### 2.4. Statistical Analysis

GraphPad Prism 8.0 (GraphPad. Software, San Diego, CA, USA) was employed for the statistical analysis. The Mann–Whitney *U* test was applied to determine intergroup differences. Receiver operating characteristic (ROC) curve analysis was performed in order to evaluate the diagnostic ability of tests to discriminate between groups. The area under the curve (AUC) was used to assess the discriminating ability of a test. The optimal cut-point value was determined using the Youden index (J), which was calculated as:

Maximum sensitivity+specificy−1

A *p*-value of less than 0.05 was considered significant.

## 3. Results

**Figure 1:**
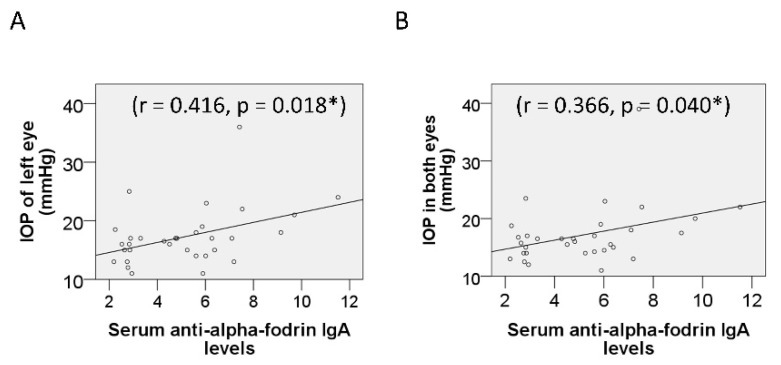
Correlation between intraocular pressure (IOP) and serum markers. The serum anti-α-fodrin IgA antibody level showed correlation with IOP in the left eye (**A**) and with mean IOP in both eyes (**B**). * Statistically significant.

A total of 42 participants who had undergone complete ophthalmologic examinations at the Ophthalmology Department of Kangnam Sacred Heart Hospital, Korea, were enrolled in this study. Three participants were excluded because of hemolysis, and one participant was excluded because of a history of trabeculectomy. Among the remaining 38 participants, 21 were assigned to the glaucoma group and 17 to the control group. The demographic data of the participants are presented in Table 1. There was no significant difference in age or sex between the control and glaucoma group or the NTG and HTG group. When diagnosing glaucoma, the central corneal thickness was taken into consideration. One patient had a very thin (479 microns) cornea, although his IOP was 13 mm Hg; therefore, the corneal thickness was not considered to affect the diagnosis. Among the patients with HTG, no patient had a cornea thicker than 600 microns. Seventeen patients had bilateral and four had unilateral glaucoma. Of these four, three had NTG and one had HTG; three patients had glaucoma in their left eyes. This study included one patient with myopia higher than −6.0 D (−7.75 and −10.0 in both eyes). He showed progression, not of myopia, but of NTG. The IOP in the HTG group was higher than in the NTG group (*p* = 0.001). IOP in the left eye and the mean IOP in both eyes showed a correlation with serum anti-α-fodrin IgA levels (r = 0.416, *p* = 0.018 and r = 0.366, *p* = 0.040, respectively; Pearson correlation analysis; Figure 1). The power of this study was calculated. When the type I error was 0.05, the post-hoc power was 100% for serum MBP, 24.6% for serum HSP60, 6.8% for anti-SSA antibody, 54.6% for anti-SSB antibody, 22.0% for anti-α-fodrin IgG antibody, 6.1% for anti-α-fodrin IgA antibody, and 6.6% for ANA levels.

In the glaucoma group, serum HSP60 levels showed a correlation with mean deviation (MD) of the right eye and of both eyes (r = 0.439, *p* = 0.047, and r = 0.482, *p* = 0.027, respectively, Spearman’s rank correlation test); they showed no correlation with RNFL thickness. Other biomarker levels showed no correlation with the MD of visual fields or RNFL thickness.

### 3.1. Comparison of Control Group and Patients with Glaucoma

**Figure 2:**
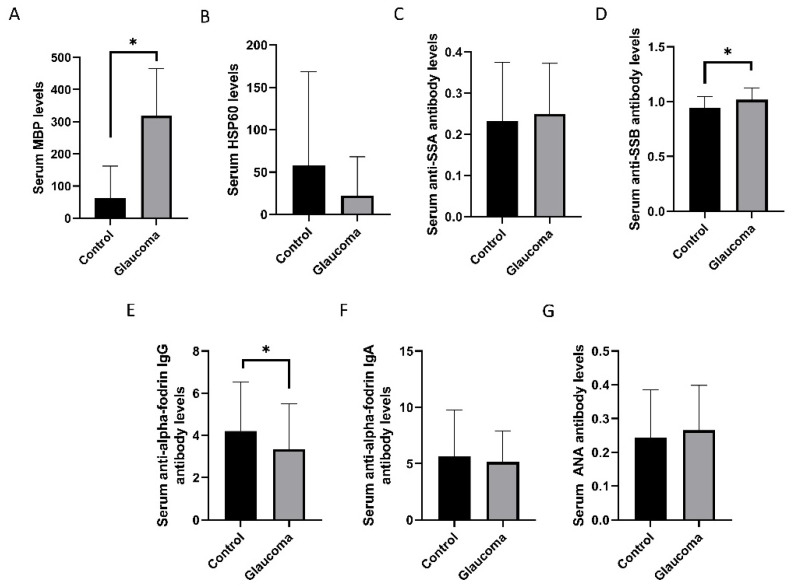
Comparison of serum markers between control and glaucoma patients. Serum myelin basic protein (MBP) levels (**A**) and serum anti-Sjögren’s-syndrome-related antigen B (SSB) antibody levels (**D**) were higher in patients with glaucoma than in the control participants. Serum levels of heat shock protein 60 (HSP60) (**B**) and serum anti-Sjögren’s-syndrome-related antigen A (SSA) antibody (**C**) were not different between the groups. Anti-α-fodrin IgG antibody levels (**E**) were lower in patients with glaucoma than in the control participants. Anti-α-fodrin IgA antibody (**F**), and anti-nucleic acid (ANA) antibody (**G**) levels were not different between the groups. * *p* < 0.05 by Mann–Whitney *U* test.

The biomarkers for discrimination between NTG and HTG were evaluated (Table 1, Figure 2). The serum MBP level was higher in the glaucoma group than in the control group (318.12 ± 146.91 pg/mL vs. 61.91 ± 100.02 pg/mL, respectively, *p* < 0.001, Mann–Whitney *U* test). The serum anti-SSB antibody index was higher in the glaucoma group than in the control group (0.94 ± 0.10 vs. 1.01 ± 0.11, respectively, *p* = 0.033; Figure 2D). The serum anti-α-fodrin IgG concentration was lower in the glaucoma group than in the control group (3.35 ± 2.13 U/ml vs. 4.21 ± 2.32 U/mL, respectively, *p* = 0.045; Figure 2E). The control and glaucoma groups showed no differences in the serum anti-SSA antibody index, serum HSP60 concentration, serum anti-α-fodrin IgA concentration, or serum ANA antibody index.

**Figure 3:**
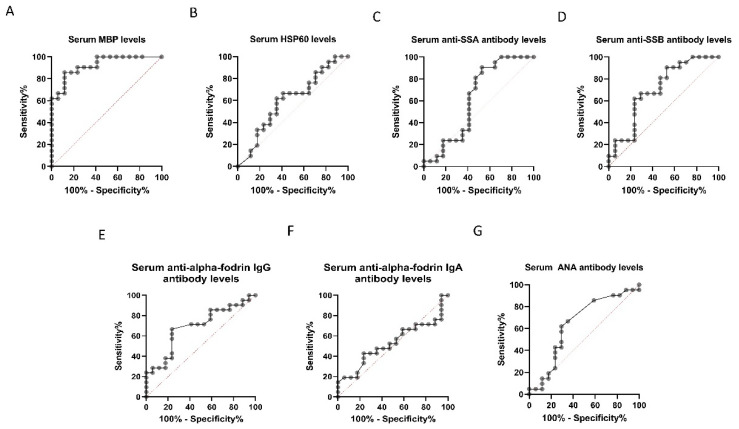
Receiver operating characteristic (ROC) curve analysis for discrimination between control participants and patients with glaucoma. ROC curves for the serum levels of myelin basic protein (MBP) (**A**), heat shock protein 60 (HSP60) (**B**), anti-Sjögren’s-syndrome-related antigen A (SSA) antibody (**C**), anti-Sjögren’s-syndrome-related antigen B (SSB) antibody (**D**), anti-α-fodrin IgG/IgA antibody (**E**,**F**), and anti-nucleic acid (ANA) antibody (**G**) are shown. The area under the curve for serum MBP concentration was the largest.

The ROC curve analysis (Table 2, Figure 3) showed the largest AUC for the serum MBP level. The sensitivity and specificity of the MBP level for discriminating between the control group and patients with glaucoma at the optimal cut-off point of 183.4 µg/µL were 85.7% and 88.2%, respectively.

### 3.2. Comparison of NTG and HTG

**Figure 4:**
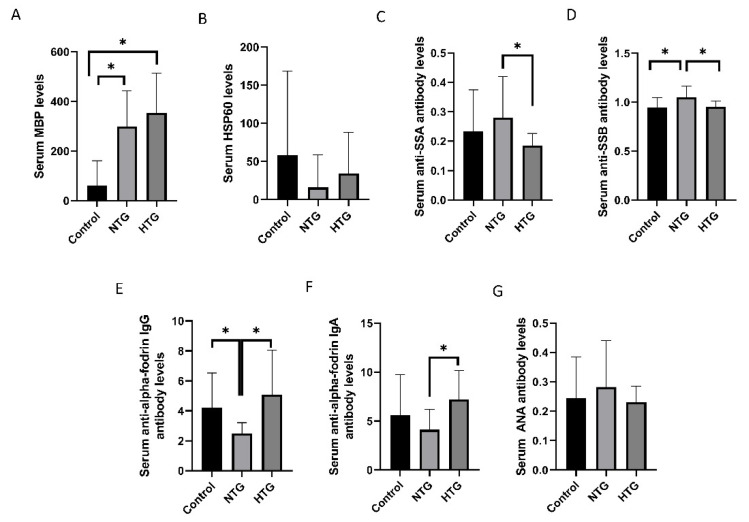
Comparison of serum markers between normal-tension glaucoma (NTG) and high-tension glaucoma (HTG). (**A**,**B**) Serum myelin basic protein (MBP) levels were higher in NTG and HTG compared to the control group, and heat Scheme 60. (HSP60) levels were not different between the NTG and HTG groups. (**C**) Serum anti-Sjögren’s-syndrome-related antigen A (SSA) antibody level in the NTG group was higher than that in the HTG group. (**D**) Serum anti-Sjögren’s-syndrome-related antigen B (SSB) antibody level in the NTG group was higher than those in the HTG group or controls. (**E**) The NTG group showed a lower serum anti-α-fodrin IgG level than the HTG and control groups. (**F**) Serum anti-α-fodrin IgA level in the HTG group was higher than that in the NTG group. (**G**) There was no difference in serum anti-nucleic acid (ANA) antibody levels between the groups. * *p* < 0.05 by Mann–Whitney *U* test.

Evaluation of the biomarkers for discrimination between NTG and HTG (Table 1, Figure 4) showed that serum MBP levels in both the NTG and HTG groups were higher than in the control group (*p* < 0.001 for both). The serum anti-SSA antibody index was higher in the NTG group than in the HTG group (0.28 ± 0.14 vs. 0.18 ± 0.05, *p* = 0.005). The serum anti-SSB antibody level was higher in the NTG group than in the control or HTG groups (*p* = 0.004 and *p* = 0.003, respectively). The serum anti-α-fodrin IgG concentration was lower in the NTG group than in the control or HTG groups (*p* = 0.002 and *p* = 0.010, respectively). The serum anti-α-fodrin IgA concentration was lower in the NTG group than in the HTG group (*p* = 0.012). The NTG and HTG groups showed no significant difference in serum MBP, HSP60, or ANA antibody levels.

**Figure 5:**
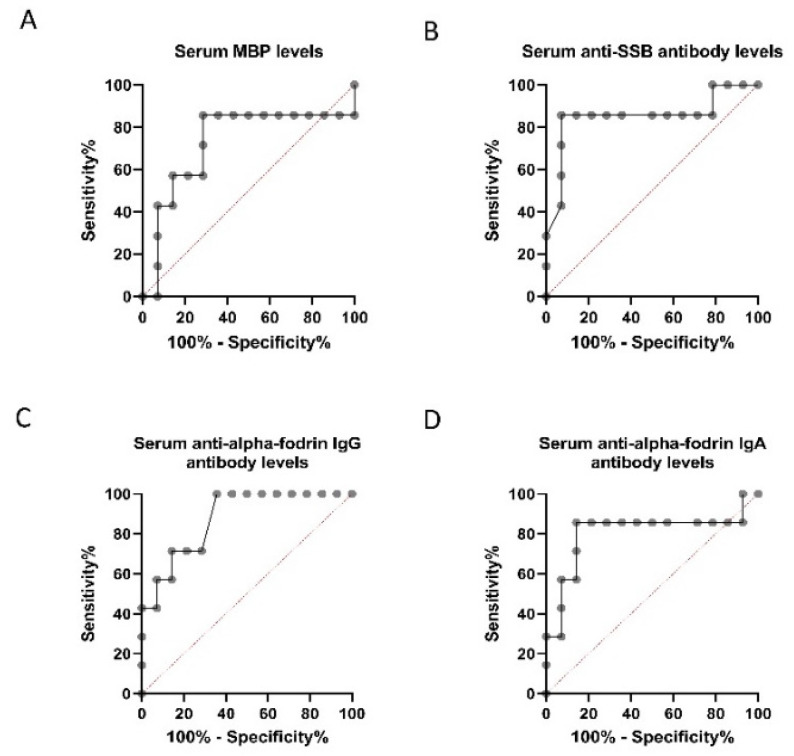
Receiver operating characteristic (ROC) curve plots for discrimination between normal-tension glaucoma (NTG) and high-tension glaucoma (HTG) patients. ROC curves for serum and myelin basic protein (MBP) concentration (**A**), anti-Sjögren’s-syndrome-related antigen B (SSB) antibody level (**B**), and anti-α-fodrin IgG/IgA antibody level (**C**,**D**) are shown. The area under the curve for the serum anti-SSB antibody level was the largest.

In the ROC curve analysis (Table 3, Figure 5), the anti-SSB antibody index showed the largest AUC for discriminating between NTG and HTG. The sensitivity and specificity of the anti-SSB antibody level at the optimal cut-off point of 0.962 were 87.5% and 92.9%, respectively.

**Figure 6:**
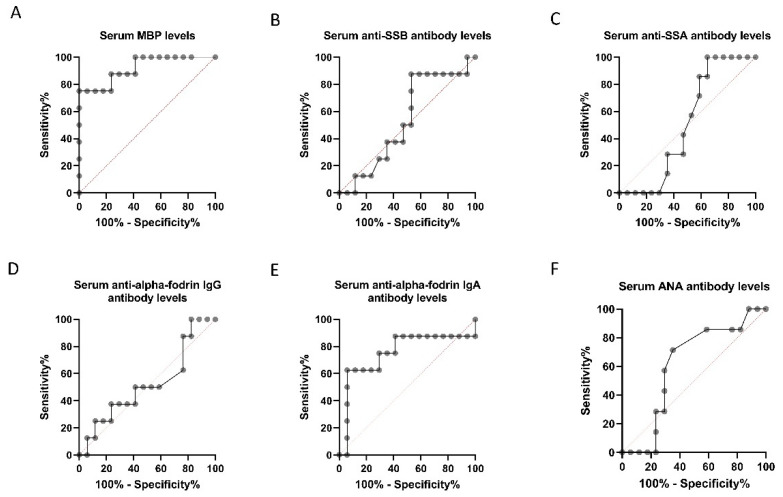
Receiver operating characteristic (ROC) curve plot for discrimination between control and high-tension glaucoma (HTG) patients. ROC curves for serum and myelin basic protein (MBP) concentration (**A**), anti-Sjögren’s-syndrome-related antigen B (SSB) antibody level (**B**), anti-Sjögren’s-syndrome-related antigen A (SSA) antibody level (**C**), anti-α-fodrin IgG/IgA antibody level (**D**,**E**) and ANA level (**F**) are shown. The area under the curve for the serum MBP antibody level was the largest.

In the ROC curve analysis (Table 4, Figure 6), the serum MBP levels showed the largest AUC for discriminating between the control and HTG groups. The sensitivity and specificity of the serum MBP level at the optimal cut-off point of 0.941 were 85.7% and 94.1%, respectively.

**Figure 7:**
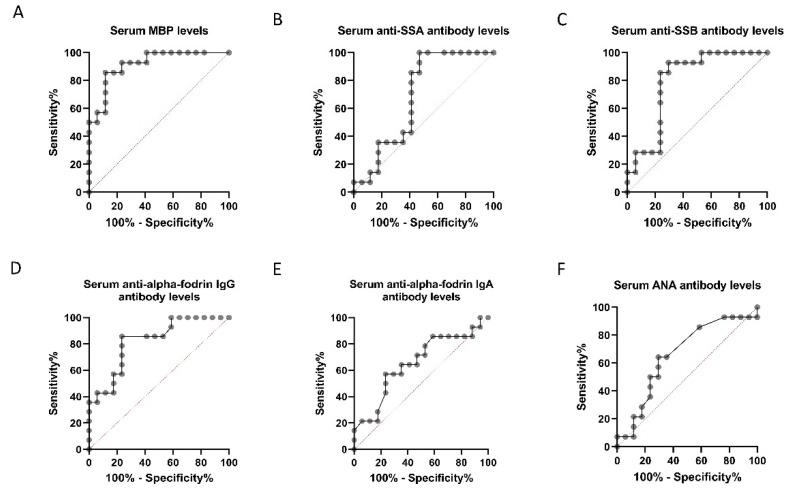
Receiver operating characteristic (ROC) curve plot for discrimination between control and normal-tension glaucoma (NTG) patients. ROC curves for serum and myelin basic protein (MBP) concentration (**A**), anti-Sjögren’s-syndrome-related antigen A (SSA) antibody level (**B**), anti-Sjögren’s-syndrome-related antigen B (SSB) antibody level (**C**), anti-α-fodrin IgG/IgA antibody level (**D**,**E**) and ANA level (**F**) are shown. The area under the curve for the serum MBP level was the largest.

In the ROC curve analysis (Table 5, Figure 7), the serum MBP levels showed the largest AUC for discriminating between the control and NTG groups. The sensitivity and specificity of the serum MBP level at the optimal cut-off point of 0.916 were 85.7% and 88.2%, respectively.

## 4. Discussion

Although the causative factors for the development and progression of glaucoma remain incompletely explored and understood, the importance of the early detection of glaucoma has been emphasized. Increased IOP is considered to be a marker for the detection of glaucoma as well as a major risk factor. However, the IOP level is not solely responsible for the development and progression of glaucoma. Although the most common form of glaucoma in the West is HTG, NTG constitutes the majority (52–92%) of glaucoma cases in Asia [14,18]. Since HTG presents with a high IOP, it is easy to detect with IOP measurements, but NTG shows a normal IOP, making it difficult to detect. In patients with NTG, mechanisms other than IOP elevation may be involved in the onset and progression of glaucoma [15,16,27], and the factors that participate in these mechanisms may be useful as markers.

In this study, the serum level of MBP, a trans-membrane protein that plays an important role in the myelination process in the central and peripheral nervous system [28], was higher in the patients with glaucoma. An increased serum MBP level is an indicator of brain damage or demyelination [29]. Moreover, anti-MBP antibody reactivities were found in patients with HTG but not in the control group [27]. The high serum MBP level in patients with glaucoma in the present study suggests optic nerve damage or brain damage and can be used as a marker to distinguish between control participants and patients with glaucoma. Serum MBP level can be used as a screening test for glaucoma. HSP60 in the matrix of mitochondria is a mitochondrial chaperonin, which is essential for the folding and assembly of newly imported proteins [30,31]. While HSP60 is released by apoptotic and necrotic central nervous system cells, it modulates apoptosis in the central nervous system [31,32]. HSP60 expression is upregulated in the glaucomatous retina and optic nerve head [33]. The present study showed no difference in serum HSP60 levels between the control and glaucoma groups. The SD of serum HSP60 level was large in control group because serum HSP60 levels can be higher for other reasons including inflammation, atherosclerosis and osteoporosis [34]. However, in this study, serum HSP60 levels in patients with glaucoma showed a correlation with clinical severity, including the MD of VFs and RNFL thickness, whereas serum MBP levels showed no correlation with clinical severity. Serum MBP levels may help to detect glaucoma and cannot be used to determine the glaucoma severity. However, serum HSP60 levels cannot be used to detect glaucoma but can be used to determine the glaucoma severity in patients with glaucoma only. Several studies have reported that not only are HSPs among the most prominent autoantigens in patients with glaucoma, but also that the serum titers of HSP antibodies are elevated in many patients with glaucoma regardless of the IOP level [16,35]. Further studies are necessary to evaluate serum anti-HSP60 antibody levels in the context of glaucoma.

Despite numerous efforts to determine the relationship between glaucoma and autoimmunity, studies have been unable to clarify whether the latter is merely epiphenomena or causative. In this study, serum anti-α-fodrin IgG antibody levels were lower in patients with glaucoma. α-Fodrin is an intracellular, actin-binding, organ-specific protein of the cytoskeleton and plays a crucial role in maintaining structural integrity in mammalian cells [36]. Antibodies against α-fodrin are associated with Sjögren’s syndrome [37] and neurodegenerative diseases [38]. Fodrin is thought to be involved in the pathogenesis of neurodegenerative diseases. α-Fodrin is a target of caspase-3 and is cleaved by caspases at the early stages of apoptosis, leading to structural rearrangements [39]. In the optic nerve affected by glaucoma, apoptosis of nerve cells occurs, resulting in a decrease in the amount of α-fodrin, which may lead to a decrease in the autoantibodies against it. Serum anti-SSA antibody, anti-SSB antibody, and anti-α-fodrin IgA antibody levels were not different between the control group and patients with glaucoma in this study, and these results may account for the difference between glaucoma and Sjögren’s syndrome. In Sjögren’s syndrome, the levels of autoantibodies, including ANA, anti-SSA/Ro, anti-SSB/La, anti-α-fodrin, and HSP60 are high. Anti-SSA/Ro are the most commonly seen in autoimmune diseases, such as systemic lupus erythematosus (SLE), Sjögren’s syndrome/SLE overlap syndrome, subacute cutaneous lupus erythematosus, neonatal lupus, and primary biliary cirrhosis [40]. Sensory peripheral neuropathy in Sjögren’s syndrome is associated with the presence of anti-SSB and anti-SSA antibodies [41]. Cerebrospinal fluid anti-SSA autoantibodies could serve as biomarkers for Sjögren’s syndrome-related central nervous system involvement [42]. The presence of anti-SSA/Ro antibodies may be associated with anti-aquaporin-4 antibody positivity in neuromyelitis optica spectrum disorder [43]. However, the role of anti-SSB and anti-SSA antibodies in optic nerve damage is still being debated. It is possible that immune response and autoantibody production develop as a consequence of these diseases. In addition, damage to retinal ganglion cells due to glaucoma can induce an autoimmunity imbalance. Further studies are required to determine whether autoantibody up-regulation is a cause or consequence of glaucoma. In this study, the serum anti-α-fodrin IgA showed a correlation with IOP in the left eye. This may be because this study involves more patients with unilateral glaucoma in the left eye.

We also compared biomarker levels between the NTG and HTG groups. In comparison with the control group, both NTG and HTG groups showed higher serum MBP levels. However, there was no difference between the NTG and HTG groups. These results suggest that a high serum MBP level indicates optic nerve damage. The serum anti-SSA antibody and anti-SSB antibody levels were higher in the NTG group than in the HTG group, whereas serum anti-α-fodrin IgG and IgA antibody levels were lower in the NTG group than in the HTG group. These differences in autoantibody repertoires suggest that autoimmunity mechanisms might be involved even in NTG [44]. Furthermore, they suggest that NTG and HTG are different disease entities. The ROC curve was used to determine whether the disease could be discriminated based on these biomarkers. In the ROC analyses, serum MBP level was the best biomarker for discriminating between controls and patients with glaucoma, and serum anti-SSB antibody was the best biomarker for discriminating between NTG and HTG. Blood tests for these biomarkers may be useful for diagnosing glaucoma and discriminating between NTG and HTG.

This study has several limitations, with the primary limitation being the small sample size. Patients with other diseases affecting the biomarker were excluded from this study, but there might be other confounding factors that may affect serum biomarker levels. A prospective large cohort study is required for a more comprehensive analysis of diagnostic, prognostic, and therapeutic autoantibodies in patients with glaucoma. However, this study is significant because it suggests the role of blood biomarkers in screening for glaucoma and in distinguishing NTG from HTG. Moreover, it may provide the key difference between NTG and HTG. In addition, the changes in antibody concentration in response to treatment should be analyzed in future studies.

## 5. Conclusions

The controls and the NTG and HTG groups showed differences in specific serum biomarker levels, suggesting that the pathogenesis in the NTG and HTG groups may be different. The specific serum biomarkers identified in this study may help diagnose glaucoma with further validation. The most effective biomarker for discriminating between control participants and patients with glaucoma was the serum MBP level with the cut off value of 183.4pg/ml, while for discriminating between patients with NTG and HTG it was the serum anti-SSB level with the cut off value of 0.962. 

## Figures and Tables

**Table 1:** Serum antibody levels measured by enzyme-linked immunosorbent assay (ELISA).

Variables	Control (n = 17)	Glaucoma (n = 21)			*p*-Value between Glaucoma and Control	*p*-Value between NTG and HTG
		Total (n = 21)	NTG (n = 14)	HTG (n = 7)		
Sex (Female: Male)	9:8	15:6	9:5	6:1	0.209 *	0.314
Age (years)	41.18 ± 16.12	48.86 ± 11.93	52.00 ± 10.23	42.57 ± 13.37	0.121	0.110
IOP in left eye	14.73 ± 2.49	18.38 ± 6.23	16.21 ± 3.04	23.36 ± 5.92	0.031 ^†^	0.001 ^‡^
Mean IOP in both eyes	14.73 ± 2.01	18.48 ± 5.71	16.21 ± 2.86	23.39 ± 7.07	0.011 ^†^	0.001 ^‡^
Serum biomarkers						
MBP (pg/mL)	61.91 ± 100.02	318.12 ± 146.91	299.80 ± 142.82	354.78 ± 159.37	<0.001 ^†^	0.110
HSP60 (ng/mL)	58.3 ± 110.49	22.00 ± 46.03	15.90 ± 42.51	34.20 ± 53.73	0.294	0.360
Anti-SSA antibody (Ab index)	0.23 ± 0.14	0.25 ± 0.12	0.28 ± 0.14	0.18 ± 0.04	0.209	0.012 ^‡^
Anti-SSB antibody (Ab index)	0.94 ± 0.10	1.01 ± 0.11	1.05 ± 0.12	0.95 ± 0.06	0.033 ^†^	0.007 ^‡^
Anti-α-fodrin antibody (IgG) (U/mL)	4.21 ± 2.32	3.35 ± 2.13	2.50 ± 0.71	5.08 ± 2.98	0.045 ^†^	0.004 ^‡^
Anti-α-fodrin antibody (IgA) (U/mL)	5.62 ± 4.12	5.14 ± 2.75	4.12 ± 2.05	7.19 ± 2.96	0.772	0.025 ^‡^
ANA antibody (Ab index)	0.24 ± 0.14	0.26 ± 0.13	0.28 ± 0.16	0.23 ± 0.05	0.136	0.443

* Pearson’s chi-squared test; ^†^ *p* < 0.05 by Mann–Whitney *U* test (comparison with total glaucoma and control group); ^‡^ *p* < 0.05 by Mann–Whitney *U* test (comparison with NTG and HTG group). NTG = normal-tension glaucoma; HTG = high-tension primary open-angle glaucoma; IOP = intraocular pressure; HSP60 = heat shock protein 60; MBP = myelin basic protein; Ab = antibody; SSA = Sjögren’s-syndrome-related antigen A; SSB = Sjögren’s-syndrome-related antigen B; ANA = anti-nucleic acid.

**Table 2:** Receiver operating characteristics curve analysis to discriminate between control and patients with glaucoma.

	AUC	95% CI	*p*-Value	Cut Off Value	Sensitivity	Specificity	J-Index
MBP (pg/mL)	0.924	0.845–1.000	<0.001 *	183.4	0.857	0.882	0.739
HSP60 (ng/mL)	0.601	0.415–0.786	0.291	3.705	0.619	0.647	0.266
Anti-SSA antibody (Ab index)	0.620	0.423–0.818	0.207	0.172	0.810	0.529	0.339
Anti-SSB antibody (Ab index)	0.705	0.532–0.878	0.032 *	0.979	0.619	0.765	0.384
Anti-α-fodrin antibody (IgG) (U/mL)	0.692	0.521–0.862	0.044 *	3.209	0.667	0.765	0.432
Anti-α-fodrin antibody (IgA) (U/mL)	0.529	0.334–0.716	0.758	5.884	0.667	0.412	0.079
ANA antibody (Ab index)	0.644	0.458–0.831	0.131	0.210	0.619	0.701	0.320

AUC = area under the curve; CI = confidence interval; MBP = myelin basic protein; HSP60 = heat shock protein 60; Ab = antibody; SSA = Sjögren’s-syndrome-related antigen A; SSB = Sjögren’s-syndrome-related antigen B; ANA = anti-nucleic acid, * statistically significant.

**Table 3:** Receiver operating characteristics curve analysis for discrimination between NTG and HTG.

	AUC	95% CI	*p*-Value	Cut Off Value	Sensitivity	Specificity	J-Index
MBP (pg/mL)	0.725	0.459–0.990	0.101	353.5	0.857	0.714	0.571
HSP60 (ng/mL)	0.628	0.362–0.859	0.351	6.773	0.429	0.786	0.215
Anti-SSA antibody (Ab index)	0.837	0.642–1.000	0.014 *	0.185	0.714	0.857	0.571
Anti-SSB antibody (Ab index)	0.852	0.639–1.000	0.010 *	0.962	0.875	0.929	0.804
Anti-α-fodrin antibody (IgG) (U/ml)	0.878	0.730–1.000	0.058	2.847	1.000	0.643	0.643
Anti-α-fodrin antibody (IgA) (U/ml)	0.806	0.561–1.000	0.025 *	5.742	0.875	0.857	0.732
ANA antibody (Ab index)	0.597	0.354–0.839	0.479	0.260	0.714	0.500	0.214

AUC = area under the curve; CI = confidence interval; NTG = normal-tension glaucoma; HTG = high-tension glaucoma; MBP = myelin basic protein; HSP60 = heat shock protein 60; Ab = antibody; SSA = Sjögren’s-syndrome-related antigen A; SSB = Sjögren’s-syndrome-related antigen B; ANA = anti-nucleic acid, * statistically significant.

**Table 4:** Receiver operating characteristics curve analysis for discrimination between control and HTG.

	AUC	95% CI	*p*-Value	Cut Off Value	Sensitivity	Specificity	J-Index
MBP (pg/mL)	0.941	0.825–1.000	0.001	295.9	0.857	0.941	0.798
HSP60 (ng/mL)	0.529	0.278–0.781	0.724	3.705	0.571	0.647	0.218
Anti-SSA antibody (Ab index)	0.508	0.283–0.734	0.949	0.216	0.857	0.411	0.268
Anti-SSB antibody (Ab index)	0.517	0.278–0.756	0.899	0.928	0.714	0.470	0.184
Anti-α-fodrin antibody (IgG) (U/mL)	0.567	0.310–0.825	0.611	3.683	0.571	0.588	0.159
Anti-α-fodrin antibody (IgA) (U/mL)	0.723	0.459–0.986	0.092	7.305	0.571	0.941	0.512
ANA antibody (Ab index)	0.613	0.376–0.851	0.391	0.195	0.714	0.647	0.361

AUC = area under the curve; CI = confidence interval; NTG = normal-tension glaucoma; HTG = high-tension glaucoma; MBP = myelin basic protein; HSP60 = heat shock protein 60; Ab = antibody; SSA = Sjögren’s-syndrome-related antigen A; SSB = Sjögren’s-syndrome-related antigen B; ANA = anti-nucleic acid.

**Table 5:** Receiver operating characteristics curve analysis for discrimination between control and NTG.

	AUC	95% CI	*p*-Value	Cut Off Value	Sensitivity	Specificity	J-Index
MBP (pg/mL)	0.916	0.820–1.000	<0.001	183.4	0.857	0.882	0.739
HSP60 (ng/mL)	0.637	0.437–0.836	0.197	4.568	0.714	0.588	0.302
Anti-SSA antibody (Ab index)	0.685	0.490–0.880	0.081	0.172	1.000	0.574	0.574
Anti-SSB antibody (Ab index)	0.798	0.634–0.963	0.005	0.979	0.857	0.765	0.622
Anti-α-fodrin antibody (IgG) (U/mL)	0.821	0.674-0.969	0.002	3.207	0.857	0.765	0.622
Anti-α-fodrin antibody (IgA) (U/mL)	0.655	0.457–0.854	0.142	2.817	0.571	0.765	0.336
ANA antibody (Ab index)	0.660	0.462–0.858	0.132	0.210	0.643	0.706	0.349

AUC = area under the curve; CI = confidence interval; NTG = normal-tension glaucoma; HTG = high-tension glaucoma; MBP = myelin basic protein; HSP60 = heat shock protein 60; Ab = antibody; SSA = Sjögren’s-syndrome-related antigen A; SSB = Sjögren’s-syndrome-related antigen B; ANA = anti-nucleic acid.

## Data Availability

All the data utilized in this study are available upon request to the corresponding author.
